# Long-Term Mortality of 306,868 Patients with Multi-Vessel Coronary Artery Disease: CABG versus PCI

**DOI:** 10.9734/bjmmr/2013/3380

**Published:** 2013-04-02

**Authors:** Jimmy T. Efird, Wesley T. O’Neal, Stephen W. Davies, Whitney L. Kennedy, Lada N. Alger, Jason B. O’Neal, T. Bruce Ferguson, Alan P. Kypson

**Affiliations:** 1 East Carolina Heart Institute, Department of Cardiovascular Sciences, Brody School of Medicine, East Carolina University, Greenville, NC, USA; 2 Departmentof Public Health, Brody School of Medicine, East Carolina University, Greenville, NC, USA; 3 Center for Health Disparities Research, Brody School of Medicine, Greenville, NC, USA; 4 Departmentof General Surgery, University of Virginia School of Medicine, Charlottesville, VA, USA; 5 Department of Anesthesia, Critical Care, and Pain Medicine, Beth Israel Deaconess Medical Center, Harvard Medical School, Boston, MA, USA

**Keywords:** CABG, PCI, survival, long-term

## Abstract

**Background:**

Several randomized controlled trials (RCT) have reported no difference in long-term mortality between coronary artery bypass grafting (CABG) and percutaneous coronary intervention (PCI). The purpose of this pooled observational analysis was to compare recent retrospective studies examining long-term survival of patients with multi-vessel coronary artery disease undergoing CABG and PCI.

**Methodology:**

We searched Medline for observational studies comparing long-term (>1 year) survival between CABG and PCI for the treatment of multi-vessel coronary artery disease over the past 10 years.

**Results:**

Eight studies met inclusion criteria. A total of 306,868 patients (155,502 CABG; 151,366 PCI) were identified. Follow-up ranged from 1 to 8 years. Mantel-Haenszel combined hazard ratios (HR) for mortality demonstrated a protective benefit of CABG compared with PCI (HR=0.77, 95%CI=0.75–0.79).

**Conclusion:**

These findings suggest a long-term survival advantage for CABG compared with PCI in patients with multi-vessel coronary artery disease.

## 1. INTRODUCTION

Coronary artery disease (CAD) is the cause of 1 in 6 deaths in the U.S. with 785,000 Americans experiencing a new myocardial infarction (MI) and 470,000 experiencing a recurrent event annually [1]. Current therapies for CAD are aimed at reducing myocardial oxygen demand and improving blood flow to poorly perfused myocardium [2].

Revascularization can be achieved with either percutaneous coronary intervention (PCI) or coronary artery bypass grafting (CABG). Both methods provide acceptable symptomatic relief. PCI is an alternative to CABG for patients with clinically stable CAD that do not have left main disease and also in cases of acute coronary syndrome (ACS) and/or MI. The usefulness of PCI is less certain in patients with multi-vessel CAD [3].

Several randomized controlled trials (RCT) have compared long-term outcomes between CABG and PCI in patients with multi-vessel CAD [4–6]. These studies reported no difference in long-term survival between CABG and PCI. However, RCTs often have narrow selection criteria resulting in limited external validity. The purpose of this analysis was to compare long-term survival of CABG versus PCI by examining retrospective studies and to contrast the results with current RCTs.

## 2. MATERIALS AND METHODS

A Medline search from October 2002 to October 2012 included the following keywords: ‘percutaneous coronary intervention’, ‘coronary artery bypass grafting’, ‘comparison’, ‘multi-vessel’, ‘left-main coronary artery’, ‘stent’, ‘CABG’, and ‘PCI’. Reference lists of articles were reviewed for additional papers. Additionally, we performed a manual search of the table of contents of journals known to publish relevant content and contacted key researchers in the field to inquire about manuscripts in-press.

Inclusion criteria included the following: 1) Studies published in English with full text available; 2) Retrospective comparisons between CABG and PCI; 3) > 1 year of follow-up; and 4) multi-vessel CAD. Exclusion criteria included: 1) ACS/MI within 24 hours of intervention; 2) left main coronary artery disease (LMCA); 3) RCTs; and 4) studies with the main goal of a specific subpopulation comparison. Review articles, editorials, and other non-peer reviewed manuscripts or abstracts were excluded. Studies also were assessed for scientific rigor (e.g., peer reviewed, impact factor for journal), inclusion of relevant independent and outcome-related variables, appropriate sample size, statistical heterogeneity of results, validity (internal, external), similarity of hypotheses across studies, evidence of a sufficient knowledge base for statistical integration, and consistency of evidence. A scoping review was undertaken as a means of refining the specific question for the systematic integration of the studies [7]. Quality of manuscripts was assessed using a domain-based evaluation [8]. We considered studies of greater than 1 year to be “long-term.”

Two reviewers independently conducted literature searches and discrepancies were resolved by consensus. Abstracts of relevant articles were evaluated for inclusion in this study. Data concerning study characteristics and comorbid conditions were recorded.

Source information was tabulated for all studies including, publication year, country of data collection, report type, and language in which the study was published. Statistical analyses were conducted using SAS^®^ Software (Version 9.3, Cary, NC). Hazard ratios (HR) and 95% confidence intervals (CI)were individually plotted to visualize differences between studies. Summary HRsand95%CIswere computed by adapting standard Mantel-Haenszel (M-H) methods for determining weighted log-normal relative effect measures [9]. Homogeneity of HRs was tested using Tarone’s approximate score method [10]. Although unpublished studies were not included in the current analysis, we collected basic information on these studies when available to help determine possible publication bias (file-drawer effect). A methods moment was employed to assess the sensitivity of results to hypothetical unpublished studies [11].

## 3. RESULTS AND DISCUSSION

### 3.1 Results

**Fig. 1:**
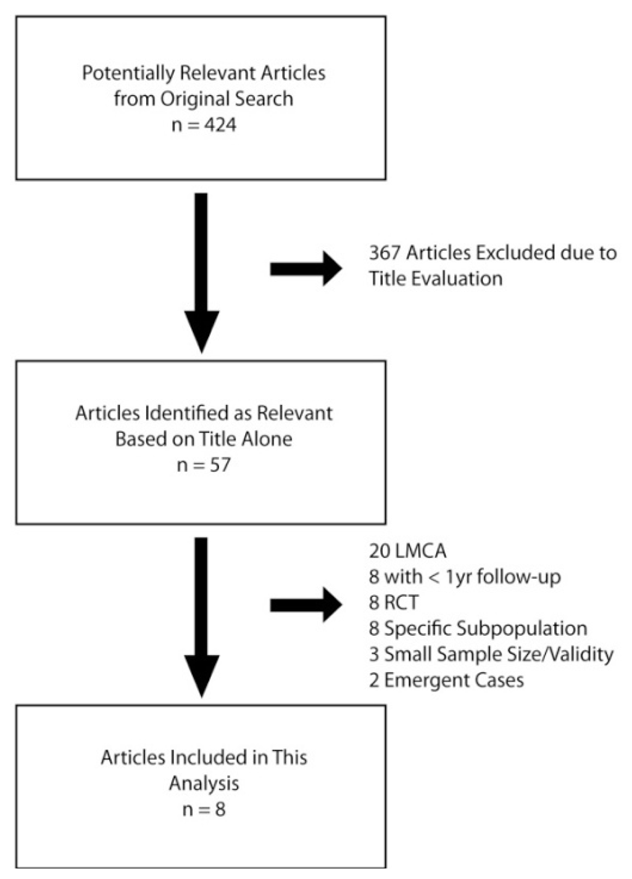
Searchcriteria LMCA=left main coronary artery; RCT=randomized controlled trial.

A total of 424 relevant articles were identified. Fifty-seven articles were selected for further review based upon their title. Of these, 8 articles met final inclusion criteria. The selection process is outlined in Fig. 1. Study and patient characteristics and are shown in Tables 1 and 2, respectively.

Our null hypothesis that heterogeneity across studies reflects random fluctuation was not rejected at the α-level = 0.05 level of statistical significance. Publication bias was not considered to be important based on a methods moment analysis.

**Fig. 2:**
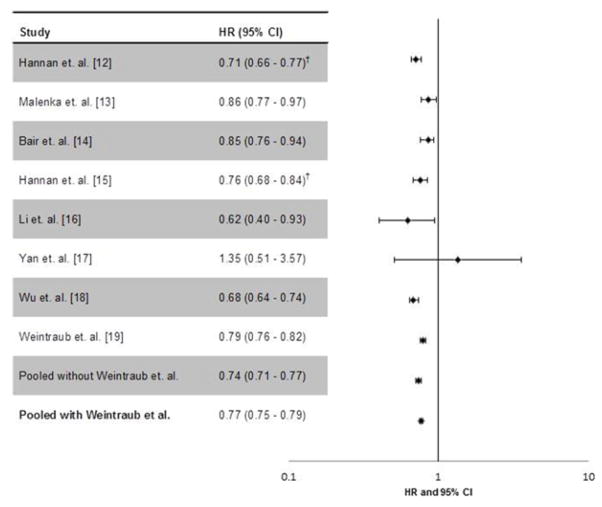
Forest plot reflecting effect of CABG on total mortality compared with PCI Forest Plot reflects HRs and 95%CIs for each individual study included in this meta-analysis. ^†^Estimates were pooled from within the study. HR < 1 reflects a survival benefit for CABG; HR > 1 reflects a survival benefit for PCI. CABG=coronary artery bypass grafting; CI=confidence interval; PCI=percutaneous coronary intervention; HR=hazard ratio.

The pooled data included 155,502 (50.7%) CABG patients and 151,366 (49.3%) PCI patients. The summary M-H HR was 0.77 (95%CI=0.75–0.79) (Fig. 2). Exclusion of the largest study did not substantively change results (HR=0.74, 95%CI=0.71–0.77).

### 3.2 DISCUSSION

To our knowledge, this is the first analysis to evaluate the long-term survival of patients who underwent either CABG or PCI for the treatment of multi-vessel CAD from observational studies. Our results suggest that patients undergoing CABG have increased long-term survival compared with PCI.

Generally, RCTs are considered to be the best evidence when comparing the efficacy of treatment groups while retrospective studies, which are prone to recall and selection bias, are believed to be less convincing than prospective trials [20]. However, RCTs have known barriers to patient participation and also may not generalize to the population at-large due to narrow selection criteria [21,22]. For example, the poor, minorities, females, and the elderly often are underrepresented in many clinical trials [23].

Our results are consistent with 2 recent clinical trials. In the SYNTAX trial, 3-year major adverse cardiac and cerebrovascular events (MACCE) remained significantly increased for PCI compared with CABG in patients with multi-vessel CAD [4]. However, this study did not specifically examine mortality as the primary endpoint. The FREEDOM trial, which enrolled 1,900 patients at 140 international centers, reported reduced mortality among CABG-treated patients compared with PCI [24]. In contrast to the current analysis, the FREEDOM trial was limited to diabetic patients.

#### 3.2.1 Limitations

We did not have access to the source data for any of the studies used in this analysis. The analysis was based on effect sizes and confidence intervals obtained from published studies. Accordingly, we were unable to use random effect models for pooling the data [25].

Another limitation is that the hazard rates between groups were not parallel for all studies included in our analysis. For example, PCI was observed to have better outcomes in the first 30 days in one study, however, a survival advantage in favor of CABG was observed for the remaining 4 years of the study [19].

Analyses combining studies cannot improve the quality or reporting of the original studies [26]. Variability between studies in reporting preoperative comorbidities, demographics, and outcome measures limits the comparative ability of such analyses. Selection bias also may be a limitation of this pooled analysis. One study in our analysis, comprising 62% of the overall sample, potentially may have marginalized the impact of smaller studies [19]. Furthermore, the heterogeneity of some studies included may have limited the interpretability of our findings. However, our results remained statistically significant after excluding this study. An inherent weakness of the current study is that patients were clinically different before their respective revascularization procedure.

No differentiation was made between patients receiving drug-eluting stents (DES) and bare metal stents (BMS). DES were introduced in 2003 and their use peaked to account for 90% of PCI procedures in 2005 [27]. The studies used in this analysis were conducted during different time periods in which this technology may have varied. Additionally, we were unable to separate cardiac mortality from total mortality due to inconsistencies in the reporting of this variable across studies.

Some of the included studies were very large and their confidence intervals were narrow, making almost all differences statistically significant. However, the smallest study was not statistically significant and was the only study with a HR > 1. Because statistical significance is influenced by sample size, the results of a large study can be statistically significant without being clinically important and vice versa. The methods that we used did not distinguish between statistical and clinical significance.

## 4. CONCLUSION

Results from this pooled analysis of observational studies suggest that CABG is associated with increased survival compared with PCI for patients with multi-vessel CAD. Recent RCTs also have observed similar findings in specific populations (e.g., diabetics and patients with high SYNTAX scores). A motivation for our study was that RCTs typically are conducted in highly selected populations. Thus, it is important to understand how mortality would compare for the general population of people who receive CABG and PCI. However, we still recognize that results from RCTs represent a higher level of evidence than observational studies due to the ability of randomization to render compared groups similar at baseline. Future RCTs comparing revascularization procedures may benefit by selecting a broader range of patients more reflective of the general CAD population, and including minorities, persons of low socioeconomic status, and females. Furthermore, our study highlights the limitation of completely relying on RCTs to evaluate the efficacy of cardiovascular interventions.

The degree to which inferences may be drawn depends on both internal and external validity. Internal validity for a RCT is based on the integrity of methods used to select study participants, collect information, and conduct analyses, and is a building block for external validity [28]. Apart from sampling error, RCTs strive to select participants in such a manner that differences between the index and reference groups are attributed only to the hypothesized effect under investigation [28]. On the other hand, a study is externally valid if unbiased inferences can be drawn beyond the extent of the study population examined. The pooled results of the observational studies used in this current analysis, which included patients with varying CAD severity and demographic backgrounds, satisfy external validity criteria. In contrast, this may be limited in some RCTs.

## Figures and Tables

**Table 1:** Studies included and characteristics

Study	Year	Single/multi-center	Study length (years)	Mean follow-up PCI/CABG (Years)	N	CABG(%)	PCI (%)
Hannan et al.[12]	2005	Multi-Center	3	1.60/1.93*	59,314	62.7	37.3
Malenka et al.[13]	2005	Multi-Center	7	3.61†	14,493	70.4	29.6
Bair et al.[14]	2007	Single	5	6.8/7.3	6,369	71.9	28.1
Hannan et al.[15]	2008	Multi-Center	> 1	1.56/1.59	17,400	42.7	57.3
Li et al.[16]	2009	Single	3	2.76/3.24*	3,720	50.7	49.3
Yan et al.[17]	2009	Single	2	NR	1,309	54.2	45.8
Wu et al.[18]§	2011	Multi-Center	8	8.0*†‡	14,470	50.0	50.0
Weintraub et al.[19]	2012	Multi-Center	5	2.63/2.82	189,793	45.4	54.6

§ Patients in CABG and PCI groups were matched based on propensity scores.

* Median value.

† Mean follow-up reported for the entire study.

‡ Non-censored follow-up reported.

CABG=Coronary Artery Bypass Grafting; PCI=Percutaneous Coronary Intervention. NR=not reported.

**Table 2:** Patient characteristics

Variable	Hannan [12]	Malenka [13]	Bair [14]	Hannan [15]	Li [16]	Yan [17]	Wu [18]	Weintraub [19]
Age	67.0/65.0†	64.5/62.2††	66.6/64.5††	66.0/65.4††	60.8/58.4††	61.4/61.4††	65.6/65.6††	74.0/74.0††
White	89.2/87.0	NR	NR	87.7/87.7	NR	NR	83.4/83.2	NR
Male	70.9/68.6	73.3/69.6	76.5/74.9	72.5/67.2	82.9/80.8	78.7/75.8	70.1/70.0	62.3/62.8
BMI	NR	NR	NR	NR	25.4/25.6	25.7/26.1	NR	NR
HTN	NR	NR	60.5/61.0	NR	65.3/61.2	58.8/68.7	NR	83.9/83.8
HLD	NR	NR	52.5/59.2	NR	44.6/38.8	181/184††	NR	NR
DM	33.2/25.3	34.4/26.3	27.7/19.7	38.2/32.7	26.7/25.1	30.3/28.8	29.0/28.7	35.8/35.8
Smoking	NR	NR	42.9/24.7	NR	29.3/26.1	31.9/32.8	NR	11.9/12.0
CHF	19.5/11.4	16.4/7.9	16.2/10.8	15.7/10.1	8.5/6.5	NR	14.5/14.0	11.2/10.8
Prior CVA	6.9/4.4	NR	NR	17.3/7.7	18.1/13.0	10.3/10.2	10.6/10.6	16.6/16.6
PVD	13.3/6.5	18.3/10.9	NR	10.7/7.0	11.7/5.9	2.5/2.7	7.2/6.9	16.4/16.4
Prior MI	25.0/27.4	44.7/48.5	NR	47.5/33.7	44.6/43.2	32.7/26.2	45.9/45.6	24.5/24.7
RF	3.4/2.2	2.9/4.8	4.0/1.6	4.2/3.7	27.8/16.0	1.02/0.99††	2.6/2.5	6.1/6.1
2 Vessel	30.7/80.4	49.6/86.2	NR	30.1/75.1	18.0/77.0	26.9/55.7	62.2/62.2	NR
3 Vessel	69.3/19.6	50.4/13.8	NR	69.9/24.9	82.0/23.0	73.1/44.3	37.8/37.8	NR

Data presented as CABG/PCI. Values are percentages unless stated otherwise.

† Median.

†† Mean.

NR=not reported; BMI=Body Mass Index; CABG=coronary artery bypass grafting; CHF=congestive heart failure; CVA=cerebrovascular accident; DM=diabetes; HLD=hyperlipidemia; HTN=hypertension; MI=myocardial infarction; PCI=percutaneous coronary intervention; PVD=peripheral vascular disease; RF=renal failure.
